# Genetic risk stratification and preventive strategies for double primary HNSCC and ESCC: a single-center cohort study

**DOI:** 10.1007/s10388-026-01187-2

**Published:** 2026-02-13

**Authors:** Sakiko Naito, Tomohiro Umezu, Hayato Yamaguchi, Takahiro Muramatsu, Yasuyuki Kagawa, Takashi Morise, Yoshiya Yamauchi, Isaku Okamoto, Masakatsu Fukuzawa, Kiyoaki Tsukahara, Masahiko Kuroda, Takao Itoi

**Affiliations:** 1 Department of Gastroenterology and Hepatology, Tokyo Medical University, 6-7-1, Nishishinjuku, Shinjuku-ku, Tokyo, 160-0023 Japan; 2 Department of Molecular Pathology, Tokyo Medical University, Tokyo, Japan; 3 Department of Otorhinolaryngology, Head and Neck Surgery, Tokyo Medical University, Tokyo, Japan

**Keywords:** Head and neck cancer, Esophageal squamous cell carcinoma, Double cancer, *ALDH2* gene, *CYP2A6* gene

## Abstract

**Background:**

Head and neck squamous cell carcinoma (HNSCC) and esophageal SCC (ESCC) share risk factors, such as alcohol consumption, smoking, and aldehyde dehydrogenase 2 (*ALDH2*) gene polymorphisms. However, the involvement of these factors in the occurrence of double primary cancers remains unclear. This study aimed to examine the risk factors for double cancers.

**Methods:**

This single-center study analyzed 113 patients with HNSCC and ESCC diagnosed between 2014 and 2022, classified into four stage-based groups: Group A (early ESCC + early HNSCC), Group B (early ESCC + advanced HNSCC), Group C (advanced ESCC + early HNSCC), and Group D (advanced ESCC + advanced HNSCC). Associations among clinical factors, Lugol-voiding lesions (LVLs), and prognosis were evaluated. Genetic analyses of ALDH2, CYP2A6, and ADH1B were performed in 20 patients, and multivariate Cox analysis included tumor stage, smoking, and body mass index (BMI).

**Results:**

Smoking was more common in Group B than in Group A (89.0% vs. 73.5%, *p* = 0.013) and was associated with Lugol-voiding lesions (*p* = 0.027). Three-year overall survival declined with advancing stage (93.3%, 74.0%, 33.3%, and 36.4% for Groups A–D; *p* < 0.001). Multivariate analysis identified advanced stage, smoking (HR = 1.9, *p* = 0.009), and low BMI (< 18.5 kg/m^2^, HR = 2.3, *p* = 0.024) as poor prognostic factors. Inactive ALDH2 correlated with drinking history, and poorly metabolizing CYP2A6 was less frequent among smokers.

**Conclusions:**

Heavy smoking was associated with the development of LVLs and poor prognosis in those with double primary cancers. *ALDH2* and *CYP2A6* may contribute to cancer risk, underscoring the importance of abstinence from alcohol and smoking in preventive healthcare.

**Supplementary Information:**

The online version contains supplementary material available at 10.1007/s10388-026-01187-2.

## Introduction

Head and neck squamous cell carcinoma (HNSCC) and esophageal SCC (ESCC) are considered examples of field cancerization, often occurring simultaneously in multiple locations [1]. Both are related to alcohol consumption and smoking, and repeated exposure to acetaldehyde and aldehyde, which are metabolic products of ethanol, is a key carcinogenic mechanism underlying the development of HNSCC and ESCC [2]. Lugol chromoendoscopy enables clinical detection of Lugol-voiding lesions (LVLs) [3], which often indicate esophageal mucosal changes. Multiple LVLs are closely associated with the inactive aldehyde dehydrogenase (ALDH) 2 genotype and field cancerization [4–7].

Acetaldehyde concentrations in vivo depend on the activity of two ethanol-metabolizing enzymes, alcohol dehydrogenase (ADH) and ALDH, which oxidize ethanol to acetaldehyde and acetaldehyde to acetic acid, respectively [8]. ALDH2 is the primary enzyme responsible for acetaldehyde metabolism, and its inactivation has been implicated in the carcinogenesis of both ESCC and HNSCC [2, 3, 5, 7]. Mutations in *ALDH2* (ALDH2^*^2Glu504Lys) reduce metabolic activity and increase the risk of developing esophageal and hypopharyngeal cancers [9, 10].

Acetaldehyde is a major carcinogen in tobacco smoke [2, 11] and may have a direct effect on the epithelium of patients with HNSCC and ESCC. However, the combined effects of alcohol, smoking, and other shared risk factors on the development of multiple dysplastic lesions remain insufficiently understood. Therefore, this study aimed to investigate the clinical, lifestyle, and genetic factors associated with the development and prognosis of patients with double primary ESCC and HNSCC.

## Methods

### Patients and study design

This single-center cohort study enrolled 113 patients newly diagnosed with HNSCC and ESCC at the Tokyo Medical University Hospital between 2014 and 2022. The sample included both a retrospective cohort (2014–2022) and a prospective cohort (2023 onward).

Early-stage ESCC was defined as tumor invasion limited to the submucosa without metastasis. Advanced ESCC included tumors invading beyond the submucosa or with metastatic spread. Early-stage HNSCC was defined as tumors limited to T1–T2 without metastatic disease spread, advanced HNSCC included tumors invading the intrinsic muscular layer or deeper or with metastatic spread, according to the 8th edition of the Union International Against Cancer Control (UICC) classification.

All patients with HNSCC underwent computed tomography (CT), magnetic resonance imaging, and positron emission tomography (PET) before treatment. Staging was performed by head and neck surgeons and radiologists using the 8th edition of the UICC TNM classification. Patients with ESCC underwent CT, PET, or upper gastrointestinal endoscopy, with staging performed by endoscopists and gastroenterologists using the same classification criteria [12]. Patients were divided into four groups based on disease stage: A, early HNSCC + early ESCC; B, advanced HNSCC + early ESCC; C, advanced ESCC + early HNSCC; and D, advanced HNSCC + advanced ESCC.

**Fig. 1:**
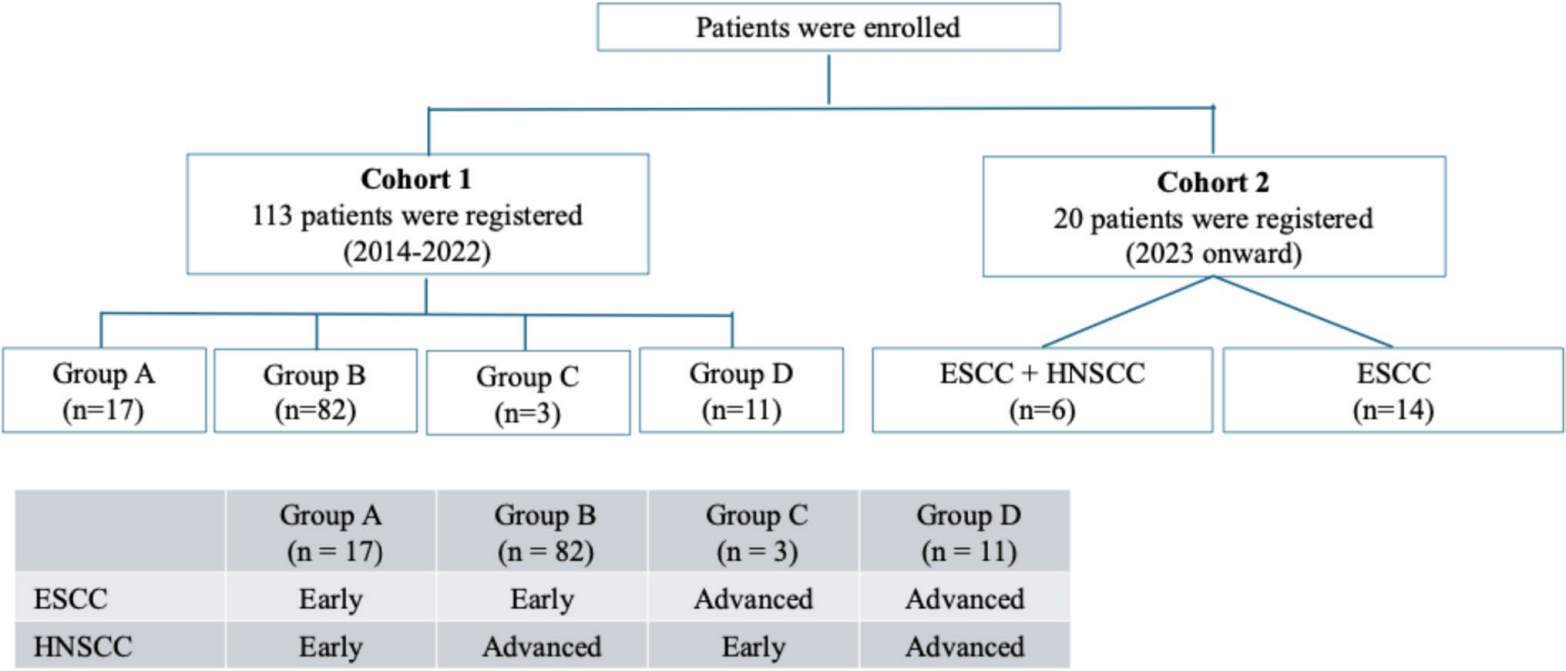
Flow diagram of patient selection

In addition, 20 patients, including six with double cancers and 14 with ESCC only, were prospectively enrolled since 2023 for genetic analyses. The objective of the prospective component was to explore the association between background factors and genetic polymorphisms, specifically *ALDH2*, *CYP2A6*, and *ADH1B*. The study design and patient flow are summarized in Fig. 1.

This study was approved by the Institutional Review Board of Tokyo Medical University Hospital (approval number: T2022-0249). Genetic analysis was approved separately by the same institution (approval number: T2022-0256), and all participants provided written informed consent (UMIN Clinical Trials Registry ID: UMIN000056557).

### Genetic analysis of *ADH1B*, *ALDH2*, and *CYP2A6*

Genomic DNA was extracted using a DNeasy Blood and Tissue kit (Qiagen) according to the manufacturer’s instructions. Briefly, samples were mixed with 200 µL of AL buffer and incubated at 56 °C for 10 min. After adding 200 µL of ethanol, samples were purified using spin columns. DNA quality and quantity were verified using Qubit 2.0 fluorometry (Life Technologies) and an Agilent 2000 TapeStation. Peripheral blood mononuclear cells were isolated from blood samples, and DNA was further assessed by gel electrophoresis for quality control.

### Sequencing

Exome enrichment of the genomic DNA was performed using the Agilent SureSelect Human All Exon V7 kit. Massively parallel sequencing was conducted using the Illumina NextSeq 2000 system. Data were analyzed using the DRAGEN Germline application, version 3.8.4, with default settings. Variant annotation was done using ANNOVAR and the following criteria were used for diagnostic filtering: (a) inclusion of known causative genes [13], (b) retention of variants with allele frequencies < 1% in gnomAD, (c) exclusion of synonymous variants, and (d) classification as “Likely” or “Pathogenic” according to the ACMG classification criteria.

Candidate variants were validated using conventional Sanger sequencing. Genetic testing was performed after obtaining written informed consent, and genetic counseling by a clinical geneticist was available upon request. The participants were categorized into three genotype groups: AA (His/His), AG (Arg/His), and GG (Arg/Arg)**,** corresponding to *ADH1B*2/*2, *ADH1B*1/*2, and *ADH1B*1/*1, respectively. *ALDH2* polymorphism GG (*1/*1) was defined as active, while GA (*1/*2) or AA (*2/*2) were defined as inactive. Complete deletion of *CYP2A6* was considered a poorly metabolizing phenotype, whereas other *CYP2A6* polymorphisms were considered functional.

### Evaluation of history of preference

Alcohol consumption was classified into five categories: non-/hardly drinkers, < 1 unit/week; light drinker, 1–8.9 units/week; moderate drinker, 9–17.9 units/week; heavy drinker, ≥ 18 units/week; and ex-drinker (1 unit = 22 g ethanol). Smoking habits were classified into three categories: nonsmoker, 0 pack-years; light smoker, < 30 pack-years; and heavy smoker, ≥ 30 pack-years [14]. The optimal cutoff value for the smoking index was determined by receiver operating characteristic (ROC) curve analysis based on Youden’s index and the area under the curve (AUC). The AUC was 0.682 (95% confidence interval [CI]: 0.578–0.785). The optimal cutoff value determined by Youden’s index was 32.5 pack-years, with a sensitivity of 82.5% and a specificity of 58.1%.

### Evaluation of LVL grades

Lugol chromoendoscopy was used to identify the squamous dysplastic lesions. LVL grades were defined as follows [8]: A, no lesions; B, 1–9 lesions; and C, ≥ 10 lesions. In this study, Grades B and C were collectively defined as “LVLs,” representing extensive mucosal field cancerization. All the images were reviewed and scored by three central review board-certified endoscopists in our hospital.

### Statistical analysis

Quantitative variables were compared using the Mann–Whitney U test, while categorical variables were analyzed using Fisher’s exact test or chi-square test, as appropriate. Overall survival (OS) was defined as the time from the first treatment to death or the last follow-up. Survival time was measured from the diagnosis of double primary cancer to death or last confirmed survival, with a cutoff in December 2024. Survival rates were calculated using Kaplan–Meier curves. Risk ratios and 95% confidence intervals (CIs) were estimated using Cox proportional hazards models. OS was analyzed using Cox proportional hazards models. Variables were selected for multivariate analysis based on clinical relevance and established prognostic factors commonly adjusted for in previous survival studies of head and neck and esophageal squamous cell carcinoma, rather than univariate statistical significance alone, to appropriately control. Multivariate Cox proportional hazards models were used to analyze OS. Furthermore, age (≥ 65 years), sex, low body mass index (BMI) (< 18.5), smoking history, and drinking history were adjusted as covariates. For the evaluation of tumor burden in synchronous double cancers, both the pharyngeal cancer stage (early vs. advanced) and esophageal cancer stage (early vs. advanced) were entered into the model. To determine whether the effect on one site was dependent on the stage of the other, an interaction term between pharyngeal and esophageal stage was included in the multivariate model (pharynx × esophagus interaction). In accordance with standard statistical practice, the interaction between pharyngeal and esophageal cancer stage was assessed by including both main effects and their interaction term in the multivariable Cox model. Statistical analyses were performed using SPSS, version 29.0 (IBM Japan, Tokyo, Japan). Statistical significance was set at *p* < 0.05.

## Results

### Comparison of characteristics between patients

A total of 113 patients diagnosed with both HNSCC and ESCC at Tokyo Medical University Hospital between 2014 and 2022 were enrolled. The group distribution was as follows: Group A, 17 patients (15.0%); Group B, 82 patients (72.6%); Group C, 3 patients (2.7%); and Group D, 11 patients (9.7%). The overall male proportion was 87.6, and the median age was 66 years (interquartile range [IQR], 59–71), with no significant differences among the groups in sex ratio or age. The incidence of HNSCC was significantly higher in the hypopharynx than in the other sites (*p* = 0.031). The number of smokers was significantly higher in Group B than in Group A (89.0% vs. 73.5; *p* = 0.013). ESCC was mostly located in the middle thoracic region in Groups A, B, and C (50, 45.1, and 33.3%, respectively), while in Group D, it was frequently located in the lower thoracic region (63.6%). The incidence of LVLs was high in all groups (Table 1).

**Table 1:** Comparison of characteristics between patients

	Group (*n* =113)				Total	*p*-value
	A (*n* = 17)	B (*n* = 82)	C (*n* = 3)	D (*n* = 11)		
Sex (male)	15 (82.4)	70 (85.4)	1 (33.3)	13 (90.9)	99	0.101
Age (years), median (IQR)	64 (54–68)	66 (59–71)	68 (56–69)	67 (61–71)	–	0.543
BMI <18.5 kg/m^2^	3 (16.7)	20 (24.3)	2 (66.6)	3 (27.3)	28	0.358
Primary tumor site of HNSCC						0.031
Pharynx	16 (94.4)	63 (76.8)	3 (100)	5 (45.5)	87	
Larynx	0	5 (6.1)	0	0	5	
Oropharynx	1 (5.6)	14 (17.1)	0	6 (54.5)	21	
Primary tumor site of ESCC						0.295
Ut	5 (27.8)	13 (15.9)	1 (33.3)	4 (36.4)	23	
Mt	9 (50)	37 (45.1)	1 (33.3)	0	47	
Lt	4 (22.2)	32 (39.0)	1 (33.3)	7 (63.6)	44	
Alcohol drinking habits						0.446
Heavy	7 (38.9)	27 (32.9)	0	2 (18.2)	36	
Moderate	6 (33.3)	25 (30.5)	1 (33.3)	3 (27.3)	35	
Light	3 (17.6)	25 (30.5)	2 (66.6)	3 (27.3)	33	
Never/rare	1 (9.1)	4 (4.9)	0	2 (18.2)	7	
Ex-drinker	0	1 (1.2)	0	1 (9.1)	2	
Smoking (pack-years)						0.061
0	4 (22.2)	9 (11)	0	1 (9.1)	14	
< 30	9 (50)	22 (26.8)	1 (33.3)	2 (18.2)	34	
≥ 30	4 (23.5)	51 (62.2)	2 (66.6)	8 (72.7)	65	
LVLs (Grade B and C)	16 (94.1)	81 (98.8)	3 (100)	10 (90.9)	110	0.368
Observation periods (months), median (IQR)	44 (39–78)	35 (19–51)	22 (11–28)	21 (13–29)	–	0.006

Data are presented as number (%), unless specified

Never drinkers, < 1 U/week; light drinkers, 1–8.9 U/week; moderate drinkers, 9–17.9 U/week; heavy drinkers, ≥ 18 U/week (1 U = 22 g of ethanol)

*IQR* Interquartile range, *BMI* Body mass index, *HNSCC* Head and neck cancer, *ESCC* Esophageal squamous cell carcinoma, *Ut* Upper thoracic esophagus, *Mt* Middle thoracic esophagus, *Lt* Lower thoracic esophagus, *LVL* Lugol-voiding lesion

### Smoking and alcohol drinking habits

History of smoking was present in 87.6% of patients and that of heavy smoking (≥ 30 pack-years), in 57.5%. The smoking rate was significantly higher in Group B (89.0%) than in Group A (73.5%) (*p* = 0.013). (Table 1) Alcohol consumption was reported in 93.8% of the patients. Heavy drinking (≥ 22 U/week) was observed in 31.8%. All 16 patients (80%) with inactive *ALDH2* had a history of alcohol consumption (*p* = 0.040), and 43.8% of them were heavy drinkers (Tables 2 and 3).

**Table 2:** Comparison of characteristics between patients with ESCC alone and HNSCC + ESCC

	HNSCC and ESCC (*n* = 6)	ESCC (*n* = 14)	Total (*n* = 20)	*p*-value
Sex (male)	66.6	71.4	70	0.831
Age (years), median	66.5	71	–	0.225
Drinking history	100	92.9	95	0.502
*Alcohol drinking habits*				
Heavy	50	28.6	35	0.357
Moderate	33.3	21.4	25	0.573
Light	16.7	42.9	35	0.260
Never/rare	0	7.1	5	0.502
*Smoking (pack-years)*				
0	33.3	28.6	30	0.831
<30	16.7	35.7	30	0.122
≥30	50	35.7	40	0.933
LVLs (Grade B and C)	100	92.9	95	0.469
ADH1B mutation (AA)	100	100	100	–
ALDH mutation (inactive)	100	71.4	80	0.143
CYP2A6 mutation	16.6	28.6	25	0.573

Data are presented as percentage, unless specified

LVLs; Grade B and C.

*ESCC* Esophageal squamous cell carcinoma, *HNSCC* Head and neck squamous cell carcinoma, *LVL* Lugol-voiding lesion, *ADH* Alcohol dehydrogenase, *ALDH* Aldehyde dehydrogenase

**Table 3:** Association between patient characteristics and genetic polymorphisms

	Inactive *ALDH2* (*n* = 16)	*p*-value
HNSCC + ESCC	37.5	0.143
ESCC	62.5	0.143
Drinking history	100	0.040
Drinking alcohol habits		0.108
Heavy	43.8	
Moderate	25	
Light	31.3	
Never/rare	0	
LVLs (Grade B and C)	100	0.040
	Poorly metabolizing *CYP2A6* (*n* = 5)	
HNSCC + ESCC	60	0.734
ESCC	40	0.831
Smoking (pack-years)		0.876
0	31.3	0.832
< 30	62.5	
≥ 30	6.3	
LVLs (Grade B and C)	80.0	0.148

Data are presented as percentage

Never drinkers, < 1 U/week; light drinkers, 1–8.9 U/week; moderate drinkers, 9–17.9 U/week; heavy drinkers, ≥ 18 U/week (1 U = 22 g of ethanol)

*ALDH* Aldehyde dehydrogenase, *HNSCC* Head and neck squamous cell carcinoma, *ESCC* Esophageal squamous cell carcinoma, *LVL* Lugol-voiding lesions

### Comparison of clinical characteristics by LVL grade

In the analysis of iodine-stained lesions, no significant differences were found in sex or age; however, heavy drinkers were more likely to have LVLs; LVLs were found in 94.7% (100/113) of all patients. LVLs were frequently observed in both HNSCC+ESCC and ESCC alone groups, with no significant difference (100% vs. 92.9%). The occurrence of LVLs was significantly higher among individuals with inactive *ALDH2* (*p* = 0.040) and higher among those with poorly metabolizing *CYP2A6* phenotype (Tables 1, 2, and 3). Smoking was a significant risk factor for the occurrence of LVLs (*p* = 0.027; Table 4).

**Table 4:** Comparison of clinical characteristics among LVL grades

	LVL grades (*n* = 113)			*p*-value
	A (*n* = 3)	B (*n* = 27)	C (*n* = 83)	
Sex (male)	3 (100)	23 (85.2)	69 (83.1)	0.725
Age (years), median	50	65	66	0.584
Alcohol drinking habits				0.245
Heavy	0	8 (29.6)	28 (33.3)	
Moderate	1 (33.3)	10 (37.0)	24 (28.6)	
Light	1 (33.3)	6 (22.2)	26 (31)	
Never/rare	1 (33.3)	3 (11.1)	3 (3.6)	
Ex-drinker	0	0	2 (2.4)	
Smoking (pack-years)				0.027
0	2 (66.6)	1 (3.7)	11 (13.3)	
< 30	1 (33.3)	9 (33.3)	23 (27.7)	
≥ 30	0	17 (63)	49 (59.0)	

Data are presented as numbers (%), unless specified

*LVL* Lugol-voiding lesion

### OS for double cancer

The median follow-up duration was 34 months (IQR, 19–53). Survival rates were compared among patients with double cancers. The 3-year OS rates were 93.3%, 74%, 33.3%, and 36.4% for Groups A, B, C, and D, respectively (*p* < 0.001), indicating that prognosis worsened when either cancer was advanced.

**Fig. 2:**
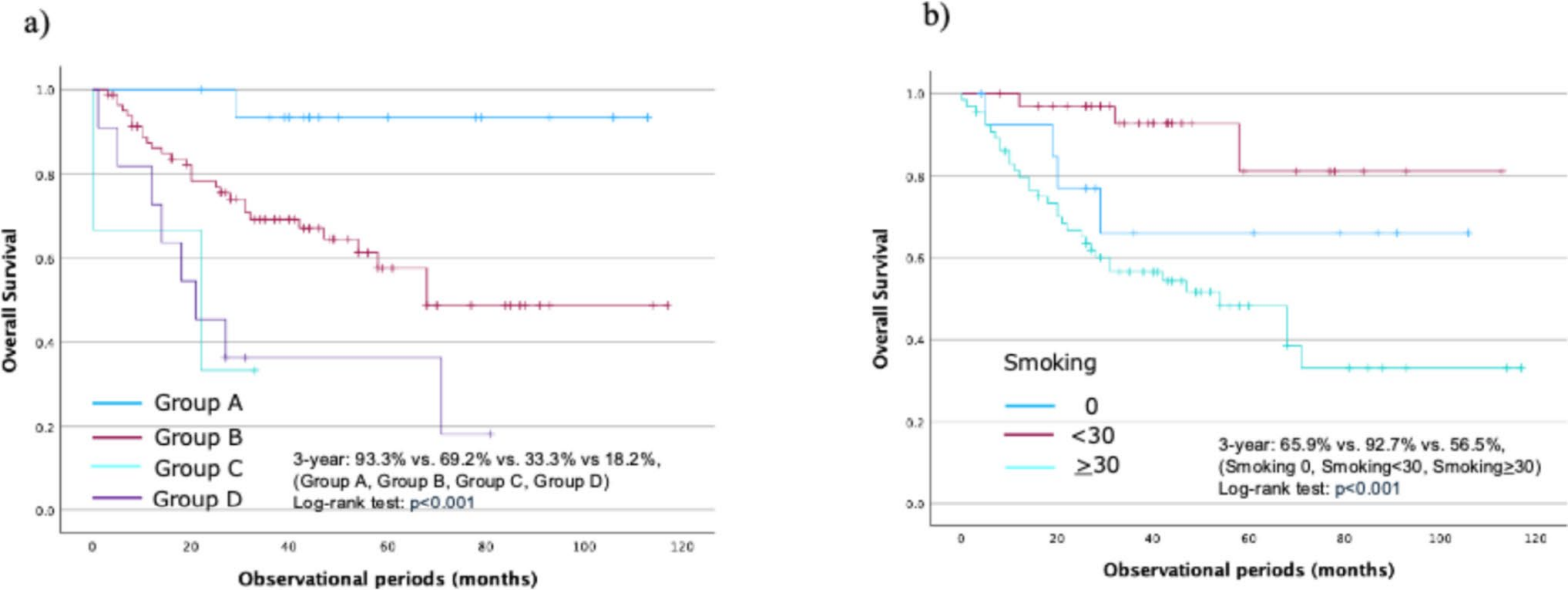
Overall survival (OS) rates of patients undergoing treatment of primary HNSCC and ESCC. **a** OS rates; **b** OS rates according to smoking levels

The 3-year OS rates of 65.9% for no smoking, 92.7% for smoking < 30 pack-years, and 56.5% for smoking ≥ 30 pack-years were associated with the presence of LVLs (*p* < 0.001) (Fig. 2).

### Univariate and multivariate analyses of prognostic factors associated with overall survival of patients with double cancer

Multivariate Cox proportional hazards analysis identified several independent prognostic factors for OS (Table 5). Sex and age were not associated with survival (*p* = 0.980 and *p* = 0.631). In contrast, low BMI (< 18.5) was an independent predictor of poor prognosis (hazard ratio [HR]: 2.356, 95% CI: 1.123–4.940, *p* = 0.023). The combined tumor-stage model, which categorized patients into four groups according to the stage of both cancers (Group A: early ESCC + early HNSCC, Group B: early ESCC + advanced HNSCC, Group C: advanced ESCC + early HNSCC, and Group D: advanced ESCC + advanced HNSCC), showed a strong association with survival. Compared with patients in Group A, those with early ESCC and advanced HNSCC (HR: 6.084, 95% CI: 0.808–45.821, *p* = 0.080), or advanced ESCC and early HNSCC (HR: 28.828, 95% CI: 2.330–356.60, *p* = 0.009) had significantly poor survival. Patients in Group D also tended to have poorer outcomes (HR: 15.940, 95% CI: 1.822–139.42, *p* = 0.012). Patients with both advanced cancers also tended to have poorer outcomes (Advanced ESCC; HR: 31.504, 95% CI: 2.580–384.696, *p* = 0.007, Advanced HNSCC; HR: 6.730, 95% CI: 0.900–50.341, *p* = 0.063) (Supplementary Table). Smoking status was significantly associated with OS overall (*p* = 0.029). Smokers showed a significantly higher risk than never smokers (HR: 2.049, 95% CI: 0.723–5.804, *p* = 0.004) in univariate analysis, whereas heavy smoker (≥ 30 pack in years) was not significantly associated with survival (*p* = 0.753). Alcohol consumption did not have a significant impact on OS (*p* = 0.578).

**Table 5:** Univariate and multivariate analyses of prognostic factors associated with overall survival of patients with double cancer

	*n* (113)	Median OS (months)	Univariate analysis				Multivariate analysis		
			HR	95% CI	*p*-value		HR	95% CI	*p*-value
*Age (years), median*									
< 65	78	40.1 (12.5–67.7)	1.0				1.0		
≥ 65	35	38.1 (12.2–64)	1.424	0.750–2.702	0.279		0.838	0.407–1.724	0.631
*Sex*									
Female	18	40.1 (12.5–67.7)	1.0				1.0		
Male	95	38.1 (10.2–64)	1.690	0.602–4.750	0.319		1.018	0.261–3.964	0.980
*BMI*									
BMI < 18.5 kg/m^2^	28	27.0 (6.2–47.8)	1.942	0.978–3.860	0.058		2.356	1.123–4.940	0.023
≥ 18.5 kg/m^2^	85	44 (16.1–71.1)	1.0				1.0		
*Alcohol drinking habits*									
Never/light	42	39.6 (12.2–67.0)	1.0				1.0		
Moderate/heavy	71	39.9 (12.6–67.2)	0.082	0.465–1.674	0.702		0.517	0.641–2.423	0.517
*Smoking (pack-years)*									
0	14	44.2 (10.4–78.0)	1.0				1.0		
< 30	33	44.1 (20.3–67.9)	0.301	0.067–1.348	0.116		0.181	0.036–0.90	0.037
≥ 30	66	36.6 (9.3–63.9)	2.049	0.723–5.804	0.004		1.246	0.351–4.21	0.758
*Group (A–D)*									
Group A	17	57.6 (29.9–85.3)	1.0				1.0		
Group B	82	38.7 (12.5–64.9)	7.753	1.055–56.989	0.044		6.084	0.808–45.81	0.080
Group C	3	18.3 (1.5–35.1)	24.183	2.172–269.273	0.010		28.828	2.330–356.60	0.009
Group D	11	39.8 (12.6–67)	19.845	2.474–159.155	0.005		15.940	1.822–139.42	0.012

*HR* Hazard ratio, *CI* Confidence interval, *BMI* Body mass index

### Genetic polymorphisms and differences in background factors between double cancers and ESCC

Among the 20 patients evaluated for ESCC, six had concurrent double cancers. Comparison between patients with double cancers and those with ESCC alone revealed that alcohol consumption was more common in the double cancer group (100% vs. 92.9%), with a high percentage of heavy smokers (50% vs. 35.7%). These 20 patients further underwent genetic analysis. In the double cancer group, the frequencies of inactive *ALDH2* and poorly metabolizing *CYP2A6* genotype were 100% and 16.6%, respectively; in the ESCC-only subgroup, the frequencies were 71.4% and 28.6%, respectively, with no statistical significance. The double cancer group exhibited the inactive *ALDH2* genotype, and the frequency of poorly metabolizing *CYP2A6* polymorphism was higher in the double cancer group. The proportion of heavy smokers was higher in the double cancer group (16.7% vs. 0%). Genetic analysis showed that the inactive *ALDH2* genotype was significantly associated with alcohol intake and the presence of LVLs in all double cancer cases (*p* = 0.040). The poorly metabolizing *CYP2A6* genotype was found in the no smoker and light smoker groups (< 30 pack-years); however, no significant difference was observed in the incidence of double cancers between these groups. Although the proportion of heavy drinkers was greater and that of the poorly metabolizing *CYP2A6* genotype was lower in the double cancer group, no significant differences were observed in gene expression between the double cancer and ESCC alone groups (Tables 2 and 3).

## Discussion

To our knowledge, this study is the first to comprehensively evaluate genetic and lifestyle-related risk factors for double primary cancers of HNSCC and ESCC in a Japanese cohort, integrating *ALDH2*, *ADH1B*, and *CYP2A6* polymorphism analysis with clinical and endoscopic findings. Our principal observation was that all patients with double cancer harbored the inactive *ALDH2* genotype, which was strongly associated with both heavy alcohol consumption and the presence of LVLs, indicating extensive background mucosal damage. These data reinforce previous evidence linking inactive *ALDH2* to elevated ESCC risk and extend its relevance to double primary malignancies. The high prevalence of LVLs in double cancer patients supports the concept of field cancerization, wherein widespread epithelial genetic instability predisposes an individual to multifocal carcinogenesis in genetically susceptible individuals. Field cancerization suggests that simultaneous exposure to alcohol drinking and smoking contributes to the development of double cancers. Alcohol drinking and smoking have been reported to damage the esophageal mucosa, and the risk of HNSCC increases with the degree of esophageal inflammation [7]. Although protective effects have been reported with the consumption of fruits and green vegetables, the risk factors associated with double cancers remain unclear. The JEC Study suggested that the *ADH1B* gene may increase the risk of secondary cancer in patients with ESCC, and that the inactive *ALDH2* and poorly metabolizing *CYP2A6* genotypes may be additional risk factors [14, 15]. *ADH1B* and *ALDH2* are involved in alcohol metabolism and delay acetaldehyde metabolism, leading to the development of double cancers [16–18]. In this study, although *ADH1B* polymorphism was not identified as a significant independent risk factor, all patients included in the genetic analysis carried the *ADH1B*2/*2 genotype (AA), which encodes a highly active enzyme variant. This form of alcohol dehydrogenase rapidly converts ethanol to acetaldehyde, a known carcinogen. In the context of inactive *ALDH2*, which slows the detoxification of acetaldehyde, the presence of *ADH1B*2/*2 further accelerates local accumulation of acetaldehyde in the upper aerodigestive tract. Previous studies have reported that the *ADH1B*2 allele increases susceptibility to esophageal and hypopharyngeal squamous cell carcinomas, particularly when combined with *ALDH2* [16–18]. The absence of genotype variation in *ADH1B* within our cohort limits our ability to draw direct conclusions regarding its contribution to double cancer risk. However, the universal presence of the high-activity *ADH1B*2/*2 genotype may suggest a genetically high-risk background for acetaldehyde-related carcinogenesis. This point underscores the importance of evaluating the combined genetic effects of alcohol-metabolizing enzymes alongside behavioral factors such as drinking and smoking in future multicenter studies. Although associations between alcohol metabolism-related polymorphisms and metachronous SCC following ESCC endoscopic resection have been reported [19, 20], specific risk factors for double cancers have not been clearly identified. This study is the first to demonstrate that heavy smoking is a prognostic risk factor and that inactive *ALDH2* and poorly metabolizing *CYP2A6* may indicate significant genetic contributors to double cancers.

### Potential mechanisms of concurrent HNSCC and ESCC development

Although field cancerization studies suggest that HNSCC and ESCC share a common pathway, the sequence of tumor development remains uncertain. Both cancers are associated with alcohol and smoking, and prior studies have reported that the risk of HNSCC is high in patients with LVLs [3]. In this study, HNSCC was significantly more common in the hypopharynx across all groups, a region prone to long-term acetaldehyde exposure due to saliva retention [2]. Groups B, C, and D had higher proportions of heavy smokers than Group A. Given that advanced cancer was associated with heavy smoking and that the hypopharynx retains saliva, it is plausible that prolonged acetaldehyde exposure promoted HNSCC development.

In addition, the high incidence of LVLs across all groups suggests that both alcohol-induced esophageal damage and chronic tobacco exposure may contribute to carcinogenesis. The present study shows that the stage of each tumor independently affects prognosis in patients with synchronous head and neck and esophageal cancers. Advanced stage in either organ was associated with worse overall survival. Notably, a significant negative interaction between esophageal and head and neck cancer stages suggests that the combined effect of dual advanced tumors is not strictly additive. Low BMI and smoking status were also associated with survival, highlighting the importance of nutritional support and smoking cessation. The poorer prognosis among heavy smokers may be partly explained by the higher proportion of advanced cancers within this subgroup. Therefore, the apparent association between heavy smoking and poor prognosis should be interpreted with caution, considering the potential confounding by tumor stage. Genetic analysis revealed that the rate of inactive *ALDH2* was significantly higher among drinkers with double cancers, and the rate of poorly metabolizing *CYP2A6* was higher among smokers with double cancers. Although the poorly metabolizing *CYP2A6* variant was found in the double cancer group, the association did not achieve statistical significance, likely due to the limited sample. In previous studies, the ALDH2*1/*2 genotype has been observed more frequently in patients with double cancer or prior cancer [21], consistent with the results of the current study. Given that acetaldehyde accumulates in the hypopharynx, preventive strategies, such as smoking cessation counseling, surveillance endoscopy, and otolaryngology examinations, are important.

### Impact of long-term exposure to acetaldehyde and aldehyde

Muto et al. reported that multiple cancerous mutations are present throughout the digestive tract in patients with HNSCC and that inactive *ALDH2* is significantly associated with LVLs [3]. In our study, the rate of inactive *ALDH2* was significantly higher among drinkers (*p* = 0.040), and the rate of the poorly metabolizing *CYP2A6* genotype was higher among non-smokers and light smokers. Nicotine metabolism is delayed in individuals with the poorly metabolizing *CYP2A6* genotype, leading to reduced smoking and a lower incidence of cancer. Nicotine is metabolized by the hepatic cytochrome P450 enzyme CYP2A6 into cotinine, which is further metabolized to form 3’–hydroxycotinine. Approximately 60% of the variation in nicotine clearance and 40% of the variation in cotinine clearance is dependent on genetic variations [22–24]. Reduced *CYP2A6* activity, common in the Japanese population [25], has been associated with shorter smoking durations and a higher likelihood of smoking cessation [26, 27], underscoring the role of *CYP2A6* polymorphisms in preventive strategies.

In patients with double cancers and LVLs, smoking was a dominant risk factor, and esophageal mucosal remodeling with age may have exacerbated smoking-induced damage [28]. Smoking cessation has been shown to reduce the incidence of carcinogenesis [29]. HNSCC+ESCC is associated with poor prognosis in the case of progression; hence, in addition to regular 6-month surveillance endoscopy [30], smoking cessation needs to be promoted as part of preventive medicine. From a clinical perspective, our findings have direct implications for risk stratification and surveillance strategies. Patients with the inactive *ALDH2* genotype and LVLs—particularly those with a heavy smoking history and low BMI—represent a subgroup at exceptionally high risk for developing HNSCC+ESCC. For such patients, we recommend intensified surveillance, including periodic chromoendoscopy with Lugol staining, coupled with preventive interventions focusing on alcohol moderation, tobacco cessation, and nutritional support. This study has several limitations. First, this was a single-center study with a small number of cases, potentially introducing bias in the expression of risk factors. In particular, the overall low proportion of heavy drinkers suggests that tobacco exposure may have played a more prominent role in the development of secondary cancers. Furthermore, heavy smoking (≥ 30 pack-years) was significantly associated with the development of severe LVLs and poor prognosis. In contrast, the apparently better survival observed among light smokers should be interpreted with caution, as it may reflect selection bias or chance variation. Second, the *ADH1B* gene was not identified in patients with double cancer. Inactive *ALDH2* and poorly metabolizing *CYP2A6* may contribute to the development of double cancer in high-risk patients. However, these findings remain preliminary owing to the limited sample size and lack of statistical significance. Given the low frequency of *CYP2A6* activity in the Japanese population and the involvement of smoking, further research is needed to clarify the association between genetic polymorphisms and carcinogenic risk to elucidate the mechanism underlying cancer development [31, 32]. Finally, the proportion of heavy drinkers in this cohort was lower than that previously reported, potentially underestimating alcohol-related risks. To validate these findings and explore whether genotype-based screening algorithms can improve early detection and patient outcomes, future large-scale, multicenter, prospective cohort studies are required.

## Conclusions

In patients with HNSCC and ESCC, heavy smoking was associated with the development of LVLs and poor prognosis. In addition, *ALDH2* and *CYP2A6* may contribute to cancer risk, suggesting that abstinence from alcohol and smoking plays a critical role in preventive healthcare.

## Supplementary Information

Below is the link to the electronic supplementary material.

Supplementary file1 (DOCX 14 kb)

## Data Availability

This study was not approved for external data sharing. Therefore, the data are not available beyond what is presented in the manuscript. Any future secondary use would require separate ethical committee’s approval.
