# Supplementary material for: Genetic risk stratification and preventive strategies for double primary HNSCC and ESCC: a single-center cohort study
Source: Esophagus. 2026 Feb 13;23(2):381–90. doi: 10.1007/s10388-026-01187-2 (PMC13038666; doi:10.1007/s10388-026-01187-2)
Supplement: Supplementary file 1 — Supplementary file1 (DOCX 14 kb) [file 10388_2026_1187_MOESM1_ESM.docx]

**Supplementary Table**: **Multivariable Cox proportional hazards model including pharyngeal cancer stage, esophageal cancer stage, and their interaction term for overall survival**

|  | **Univariate analysis** | | |  | **Multivariate analysis** | | |
| --- | --- | --- | --- | --- | --- | --- | --- |
|  | **HR** | **95% CI** | **p-value** |  | **HR** | **95% CI** | **p-value** |
| Age (years), median |  |  |  |  |  |  |  |
| <65 | 1.0 |  |  |  | 1.0 |  |  |
| >65 | 1.424 | 0.750-2.702 | 0.279 |  | 0.895 | 0.435-1.834 | 0.763 |
| Sex |  |  |  |  |  |  |  |
| Female | 1.0 |  |  |  | 1.0 |  |  |
| Male | 0.690 | 0.602-4.750 | 0.319 |  | 1.035 | 0.245-3.697 | 0.944 |
| BMI |  |  |  |  |  |  |  |
| BMI <18.5 kg/m^2^ | 1.942 | 0.978-3.860 | 0.058 |  | 2.353 | 1.115-4.966 | 0.025 |
| >18.5 kg/m^2^ | 1.0 |  |  |  | 1.0 |  |  |
| Alcohol drinking habits |  |  |  |  |  |  |  |
| Never/Light | 1.0 |  |  |  | 1.0 |  |  |
| Moderate/Heavy | 0.082 | 0.465-1.674 | 0.702 |  | 1.208 | 0.621-2.351 | 0.578 |
| Smoking |  |  |  |  |  |  |  |
| 0 | 1.0 |  |  |  | 1.0 |  |  |
| <30 | 0.301 | 0.067-1.348 | 0.116 |  | 0.167 | 0.034-0.830 | 0.029 |
| >30 | 2.049 | 0.723-5.804 | 0.004 |  | 1.220 | 0.354-4.206 | 0.753 |
| ESCC stage |  |  |  |  |  |  |  |
| Early ESCC | 1.0 |  |  |  | 1.0 |  |  |
| Advanced ESCC | 2.173 | 1.061-4.451 | 0.034 |  | 31.504 | 2.580-384.696 | 0.007 |
| HNSCC stage |  |  |  |  |  |  |  |
| Early HNSCC | 1.0 |  |  |  | 1.0 |  |  |
| Advanced HNSCC | 3.623 | 1.116-11.767 | 0.032 |  | 6.730 | 0.900-50.341 | 0.063 |
| Interaction |  |  |  |  |  |  |  |
| ESCC stage×HNSCC stage | 1.890 | 0.869-4.110 | 0.108 |  | 0.056 | 0.004-0.753 | 0.030 |

HNSCC, head and neck squamous cell carcinoma; ESCC, esophageal squamous cell carcinoma; BMI, body mass index; CI, confidence interval
